# EgmiR5179 Regulates Lipid Metabolism by Targeting EgMADS16 in the Mesocarp of Oil Palm (*Elaeis guineensis*)

**DOI:** 10.3389/fpls.2021.722596

**Published:** 2021-07-26

**Authors:** Yifei Wang, Jixin Zou, Jin Zhao, Yusheng Zheng, Dongdong Li

**Affiliations:** ^1^College of Tropical Crops, Hainan University, Haikou, China; ^2^Rubber Research Institute of Chinese Academy of Tropical Agricultural Sciences (CATAS), Haikou, China

**Keywords:** oil palm, MADS-box gene, micorRNA5179, lipid content, fatty acid

## Abstract

*EgMADS16*, one of the MADS-box transcription factors in oil palm, has a high expression level in the late fruit development of the oil palm fruit mesocarp. At the same time, it is also predicted to be the target gene of *EgmiR5179,* which has been identified in previous research. In this paper, we focused on the function and regulatory mechanism of the *EgMADS16* gene in oil palm lipid metabolism. The results indicated that the transcription level of *EgMADS16* was highest in the fourth stage, and a dual-luciferase reporter assay proved that the *EgMADS16* expression level was downregulated by *EgmiR5179*. In both the OX*EgMADS16 Arabidopsis* seeds and oil palm embryonic calli, the total lipid contents were significantly decreased, but the contents of C18:0 and C18:3 in OX*EgMADS16* lines were significantly increased. As expected, EgmiR5179 weakened the inhibitory effect of *EgMADS16* on the oil contents in transgenic *Arabidopsis* plants that coexpressed *EgmiR5179* and *EgMADS16* (OXEgmiR5179-*EgMADS16*). Moreover, yeast two-hybrid and BiFC analyses suggested that there was an interaction between the *EgMADS16* protein and EgGLO1 protein, which had been proven to be capable of regulating fatty acid synthesis in our previous research work. In summary, a model of the molecular mechanism by which miRNA5179 targets *EgMADS16* to regulate oil biosynthesis was hypothesized, and the research results provide new insight into lipid accumulation and molecular regulation in oil palm.

## Introduction

Oil palm (*Elaeis guineensis* Jacq.), which belongs to the Arecaceae family, is the most productive oil crop in the world and can accumulate up to 90% oil in the mesocarp (Bhagya et al., 2020). At present, the main oil crops that produce vegetable oils are oil palm, soybean, rape, and sunflower. They produce 79% of the total oil yields (Dyer et al., 2008). The palm oil produced by oil palm accounts for 36% of the total oil yields (Osorio-Guarin et al., 2019). Although much progress has been made on plant lipid metabolism and regulatory mechanisms *via* plant molecular biology research, it is still very difficult to obtain the ideal palm oil with the highest nutritional value or the largest yield. Presently, there are also few reports about the genes involved in the regulation of oil metabolism and related transcription factor regulation networks in oil palm (Li et al., 2020).

In plant seeds, lipids are the main form of carbon storage, constituting up to 60% of the dry seed weight (Ohlroggeav and Browse, 1995). The biosynthesis of vegetable oil is affected by many factors, including transcription factors, microRNAs (miRNAs), plant hormones, signaling molecules, and environmental factors. Several transcription factors have been found to participate in the regulation of oil biosynthesis. For example, *ZmWRI1*, which belongs to the AP2/EREBP family, could regulate FA biosynthesis and increase seed oil by up to 46% in maize seeds overexpressing ZmWRI1 without affecting the germination, seedling growth, and grain yield (Shen et al., 2010). *GmMYB73*, which belongs to the MYB family, and *GmbZIP123*, which belongs to the bZIP family, could enhance lipid contents in both the seeds and leaves of transgenic *Arabidopsis* plants (Song et al., 2013; Liu et al., 2014). In oil palm, *EgMADS21* regulates *EgDGAT2* expression and ultimately affects fatty acid accumulation in the mesocarp (Li et al., 2020). In addition, *EgWRI1-1* participated in the regulation of oil biosynthesis by interacting directly with the EgNF-YA3 protein. *EgWRKY40* interacted with *EgWRKY2* to inhibit the transcription of oil biosynthesis-related genes (Yeap et al., 2017). However, little is known about the molecular mechanism of lipid accumulation in the mesocarp of oil palm.

In our previous research, overexpressing *EgmiR5179* significantly increased the oil yield in *Arabidopsis* seeds, and one MADS-box transcription factor (*EgMADS16*) was predicted to be the target gene of *EgmiR5179* (Gao et al., 2019). In this study, we cloned full-length *EgMADS16* from the mesocarp of oil palm. Transgenic *Arabidopsis* plants and oil palm embryoids overexpressing *EgMADS16* were obtained, and the contents of oil and fatty acids were tested. Finally, transgenic *Arabidopsis* overexpressing *EgmiR5179* and *EgMADS16* was obtained, and lipid and fatty acid contents were detected to confirm the relationship between *EgMADS16*, *EgmiR5179*, and oil biosynthesis. The results depicted one pathway by which miRNAs target transcription factors and ultimately regulate lipid metabolism, providing a new strategy for lipid metabolism research in oil palm.

## Materials and Methods

### Plant Materials and Strains

Oil palm (*Elaeis guineensis* Jacq.) fruits were collected at the Chinese Academy of Tropical Agricultural Sciences in Hainan, PR China. These fruits were divided into phase 1 [30–60 days after pollination (DAP)], phase 2 (60–100 DAP), phase 3 (100–120 DAP), phase 4 (120–140 DAP), and phase 5 (140–160 DAP) according to the developmental stage (Tranbarger et al., 2011) and stored at −80°C until the next experiment. Three independent bunches were collected from three distinct individuals which at the similar stage. *Arabidopsis thaliana* plants were grown in a 23°C incubator with 16 h light/8 h dark cycles. In addition, oil palm embryoids were induced and cultured using the method in previous research (Zou et al., 2019). All strains, including *DH5α* (*E. coli*), Y187 and Y2HGold (yeast), and GV3101 (*Agrobacterium*), were maintained by our laboratory.

### RNA Extraction and Real-Time PCR Analysis

Total RNA of oil palm fruit mesocarps (0.5 g), *Arabidopsis* leaves (0.1 g), and oil palm embryoids (0.2 g) was extracted by an RNAprep Pure Plant Kit (Polysaccharides and Polyphenolics-rich; Tiangen, Beijing, China). Protoplastic RNA was isolated using TRIzol Reagent (Invitrogen, United States). cDNA was synthesized using the FastQuant RT kit (with gDNase; Tiangen, Beijing, China), and quantitative real-time PCR (qPCR) was performed with TB GreenTM Premix Ex TaqTM II (Tli RNaseH Plus; TaKaRa, Japan) on a CFX96 Touch System (Bio-Rad, United States). Each sample was set to three replicates, and the internal control for the oil palm sample was β-Actine, while that for the *Arabidopsis* sample was Actin7. In addition, all experimental steps were provided by the manufacturer, and the related primers designed by Primer Premier 5 are shown in Supplementary Table 1.

### Gene Cloning and Vector Construction

The vector pCAMBIA3301 was used for plant transformation. pGreen II 62-SK (SK) was used for protoplast transfection and the dual-luciferase reporter assay. pCAMBIA1300S containing the green fluorescent protein (GFP) gene was used for subcellular localization. The pHiS2.1, pGADT7, and pGBKT7 vectors were used for the yeast hybrid assay, and the pSPYNE and pSPYCE vectors were used for BiFC analysis. The CDS of *EgMADS16* was cloned using different primers and ligated into pGreen II 0800-miRNA (LUC), pGADT7, and pGBKT7 using a One Step Cloning Kit (Vazyme) according to the principle of homologous recombination. The CDS of *EgMADS16* without the final TGA was cloned into pCAMBIA1300S and pSPYNE. Likewise, the CDS of EgGLO1 without the final TGA was cloned into pSPYCE vectors. For the *EgMADS16*-p3301 vector, we ligated the CDS of *EgMADS16* into the pCAMBIA1300S vector, cut the 35S::*EgMADS16*::NOS terminator, and inserted the terminator into the multiple cloning site of the pCAMBIA3301 vector using a restriction enzyme. The plasmids about *EgmiR5179* were constructed and conserved in our laboratory (Gao et al., 2019). All the primers used for the vector construction are shown in Supplementary Table 2.

### Subcellular Localization in *Nicotiana benthamiana*

The subcellular localization of *EgMADS16* protein was performed in *Nicotiana benthamiana* leaves according to previous research (Jessen et al., 2015). *Agrobacterium tumefaciens* GV3101 strains harboring *EgMADS16*-p1300SGFP plasmids, which encode the MADS16-EGFP fusion protein, were incubated to an OD 600 of 0.6; after centrifugation (4,000 rpm, 10 min), they were resuspended in the equal volume buffer (0.01 M MgCl_2_, 0.01 M MES, PH5.5, and 150 μm AS) and infiltrated into the leaves of *Nicotiana benthamiana*. After incubation for 36–72 h, the GFP signals in the lower epidermis of these leaves were observed using a fluorescence inverted microscope (Nikon, TS2-LS) with an excitation wavelength of 430–510 nm. At the same time, nuclei were stained with 4', 6-diamidino-2-phenylindole (Sigma-Aldrich, United States) and observed using excitation wavelengths of 330–380 nm.

### Dual-Luciferase Reporter Assay

The 10 μg EgMADS16-LUC plasmids were transformed into 200 μl oil palm protoplasts (5 × 10^5^/ml) with 10 μg EgmiR5179-SK or SK plasmids; each sample was set to three replicates. After incubating for 14–18 h, the firefly and Renilla luciferase activities were tested using a dual-luciferase reporter gene assay kit (Promega, United States). At the same time, *EgMADS16*-LUC was transformed into wild-type or OXEgmiR5179 *Arabidopsis* protoplasts, and the dual-luciferase activities were analyzed using the same method.

### Protoplast Isolation and Transformation

The protoplast isolation and transfection of *Arabidopsis* leaves were performed as described in a previous study (Yoo et al., 2007). In addition, protoplasts of oil palm leaves were isolated according to the preparation of *Arabidopsis* leaf protoplasts, but the contents of Cellulase R10 (BR) and Macerozyme R10 (BR) were doubled. The plasmids were transferred into oil palm protoplasts using an equal volume 40% (w/v) polyethylene glycol/50 mm MgCl_2_ solution, and heat shock treatment was performed as described in previous research (Masani et al., 2014).

### *Arabidopsis thaliana* Transformation

*A. thaliana* was cultured in 16 h light/8 h dark at 23°C. *EgMADS16*-p3301 plasmids were transformed into GV3101 *Agrobacterium* and then introduced into *A. thaliana* using the floral dip method (Jin et al., 2017). Moreover, transformants were selected by 1/2 Murashige and Skoog medium containing 60 mg/L glufosinate ammonium.

### Oil Palm Embryoids Culture and Transformation

Oil palm embryoids were cultured using woody plant medium in a 28°C incubator with 12 h light/12 h dark cycles according to previous research (Zou et al., 2019). *A. tumefaciens* GV3101, which contained *EgMADS16*-p3301 plasmids, was used to infect oil palm embryoids for 15 min with an OD_600_ of 0.5. Then, these embryoids were cultured on cocultivation medium (woody plant medium supplemented with 100 mg/L cysteine and 100 μm AS) at 19°C for 2 days and on screening medium (woody plant medium supplemented with 400 mg/L timentin and 60 mg/L glufosinate ammonium) at 28°C for 4 months, and the medium was regenerated every 20 days.

### Total Lipid Extraction and Fatty Acid Analysis of the *Arabidopsis* Plants and Oil Palm Embryoids

Both the lipid and fatty acid analyses of *Arabidopsis* plants and oil palm embryoids were conducted according to previous studies (Yuan et al., 2017). In brief, 5 ml chloroform-methanol (volume ratio 2:1) was used to extract lipids, and then, lipids were dried by a Termovap sample concentrator. In addition, 10 μg C17:0 was added to the lipid samples as an internal standard. All samples were methylated by 3 ml concentrated sulfuric acid-methanol (volume ratio 1:40) at 80°C for 2 h. Then, 3 ml N-hexane and 2 ml 0.9% (W/V) sodium chloride solution were added. After centrifuging at 4,000 rpm for 5 min, the supernatant was transferred to a new tube and dried by a Termovap sample concentrator. The fatty acid methyl ester was finally dissolved in 1 ml n-hexane for GC analysis. The GC analysis was performed by the Analytical and Testing Center of Hainan University. Detailed operation parameters: the oven temperature was initially maintained at 150°C for 1 min, then increased at 8°C/min to 250°C, and then increased to 250°C and maintained for 5 min. The split ratio was 1:30, and the carrier gas was helium at a flow rate of 1.0 ml/min in constant flow mode. The injector was at 250°C, and the detector, at 230°C. And the lipid mass was calculated using the internal standard method.

### Yeast Two-Hybrid Assays

Yeast two-hybrid assays were performed using Matchmaker^™^ Gold Yeast Two-Hybrid System (Clontech, United States). According to the user manual, EgMADS16-pGBKT7 plasmids were transfected into Y2H Gold yeast strains, and the autoactivation of EgMADS16 transcription factor was tested. Then, the EgMADS16-pGBKT7 Y2H Gold yeast strains and oil palm library strains which kept by our laboratory were combined. After incubation at 30°C for 20–24 h at 45 rpm, these strains were plated on SD/Try/Leu/-His/Ade/+X-α-Gal/+Aba mediums.

### Bimolecular Fluorescence Complementation Analysis

EgMADS16-pSPYNE and EgGLO1-pSPYCE were transformed into Agrobacterium strains GV3101, respectively. And then these strains were coinfiltrated into the leaves of *N. benthamiana* using the same method as subcellular localization. Moreover, after incubation for 36–72 h, the yellow fluorescent protein (YFP) signal was observed using the excitation wavelength of 430–510 nm.

### Bioinformational Analysis

Conserved domain was analyzed in the web site.^1^ Multiple sequence alignment was carried out by Clustal Omega.^2^ Small RNA target analysis was performed using the web site.^3^ The phylogenetic tree of EgMADS16 and other MADS proteins from different species was constructed using the neighbor-joining method in MEGA6.

### Statistical Analysis

The values are the means ± SD (*n* ≧ 3). Significant differences between groups were analyzed by SPSS software using Student’s *t*-test. “^*^” represents a significant difference (*p* < 0.05), and “^**^” represents a highly significant difference (*p* < 0.01).

## Results

### Cloning and Subcellular Localization of *EgMADS16*

**Figure 1:**
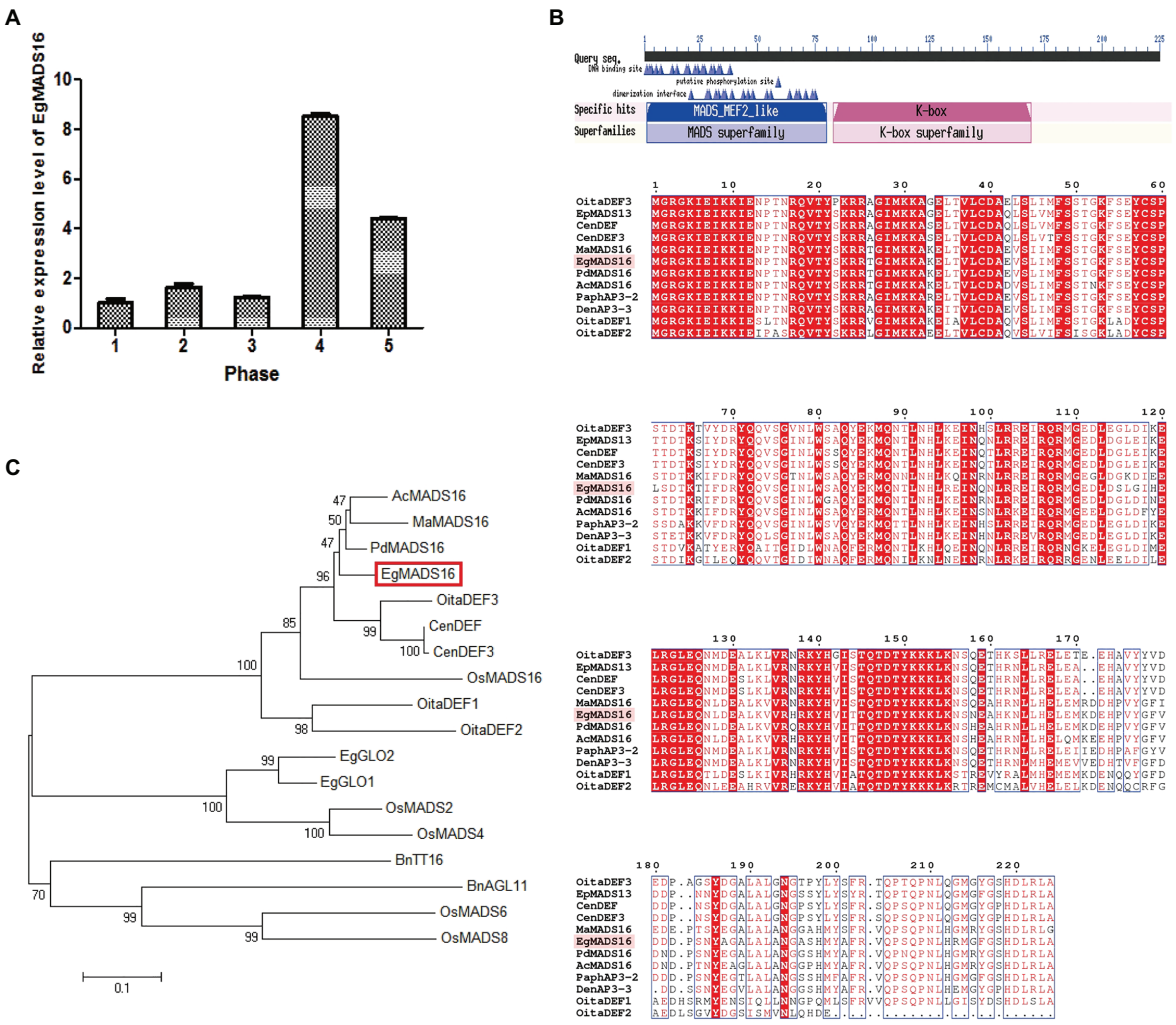
The expression patterns and sequence analysis of *EgMADS16.* **(A)** The relative expression level of *EgMADS16* in different developmental stages of oil palm fruit mesocarp. Phase 1–5: fruits 30–60 days, 60–100 days, 100–120 days, 120–140 days, and 140–160 days after pollination. The values are the means±SD (*n* = 3). **(B)** Conserved domains analysis of EgMADS16 protein. **(C)** The phylogenetic tree of EgMADS16 and other MADS proteins, including EgGLO1 (XP_010911271.1), EgGLO2 (AAQ03229.1), OsMADS2 (XP_015623988.1), OsMADS4 (XP_015640709.1), OsMADS6 (XP_015623947.1), OsMADS8 (XP_015610824.1), OsMADS16 (XP_015641661.1), AcMADS16 (XP_020082339.1), BnAGL11 (XP_013719732.1), BnTT16 (NP_001303188.1), CenDEF (ALB26780.1), CenDEF3 (AFH66787.1), OitaDEF1 (BAO00916.1), OitaDEF2 (BAO00917.1), OitaDEF3 (BAO00918.1), MaMADS16 (XP_009418316.1), and PdMADS16 (XP_008781489.1).

The mRNA of *EgMADS16* (NM_001303583) is 951 bp in length and encodes a protein of 225 amino acids (NP_001290512.1). The CDS of *EgMADS16*, which is 678 bp, from oil palm mesocarp using primers is shown in Supplementary Table 2. qPCR indicated that the expression of *EgMADS16* fluctuated from phase 1 to phase 3 and then sharply increased and reached a peak in phase 4; however, there was a gradual decline from phase 4 to phase 5 (Figure 1A). Conserved domain analysis (see footnote 1) showed that there are MADS_MEF2_like and K-box domains in the *EgMADS16* amino acid sequence (Figure 1B). In addition, we constructed a phylogenetic tree of EgMADS16 and other MADS proteins from different species using the neighbor-joining method in MEGA6, including EgGLO1 (XP_010911271.1), EgGLO2 (AAQ03229.1), OsMADS2 (XP_015623988.1), OsMADS4 (XP_015640709.1), OsMADS6 (XP_015623947.1), OsMADS8 (XP_015610824.1), OsMADS16 (XP_015641661.1), AcMADS16 (XP_020082339.1), BnAGL11 (XP_013719732.1), BnTT16 (NP_001303188.1), CenDEF (ALB26780.1), CenDEF3 (AFH66787.1), OitaDEF1 (BAO00916.1), OitaDEF2 (BAO00917.1), OitaDEF3 (BAO00918.1), MaMADS16 (XP_009418316.1), and PdMADS16 (XP_008781489.1). As shown in Figure 1C, *EgMADS16* was closest to PdMADS16 (91.56% identity), AcMADS16 (87.56% identity), and MaMADS16 (84.89% identity).

**Figure 2:**
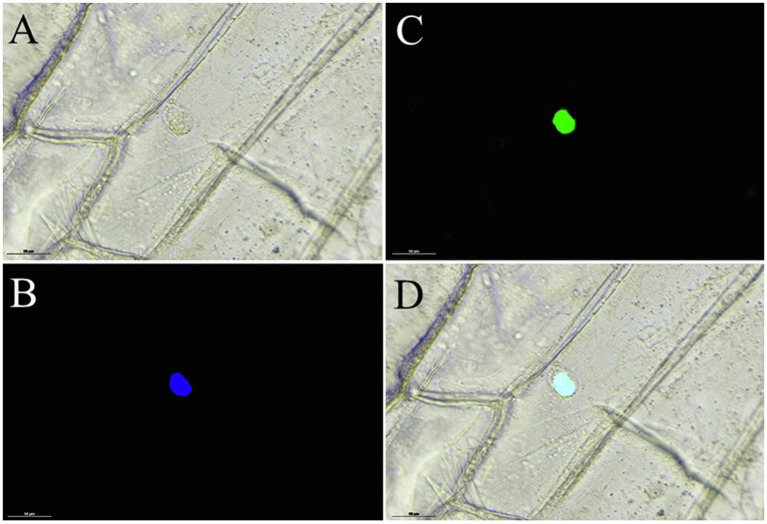
Subcellular localization of EgMADS16 protein in onion epidermal cells. **(A)** Bright light. **(B)** DAPI. **(C)** EgMADS-GFP fluorescence. **(D)** Merged image. Scale bar, 50 μm.

Moreover, to identify whether *EgMADS16* localized to the nucleus, similar to other transcription factors, we conducted a subcellular localization test in tobacco leaf epidermal cells, and the results indicated that the *EgMADS16*-GFP fusion protein was localized in the nucleus (Figure 2).

### Verification of the Target Relationship Between *EgmiR5179* and *EgMADS16*

**Figure 3:**
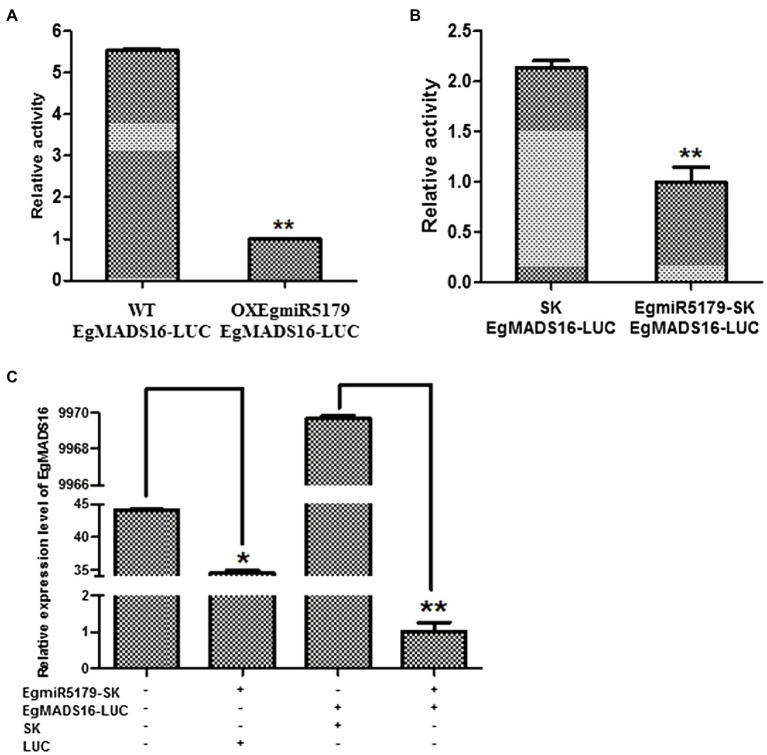
*EgMADS16* is the target gene of *EgmiR5179.* **(A)** Dual-luciferase reporter assay in Arabidopsis protoplasts. EgMADS16-LUC was transformed into wild-type and OXEgmiR5179 Arabidopsis protoplasts, respectively. **(B)** Dual-luciferase reporter assay in oil palm protoplasts. EgMADS16-LUC and SK or EgmiR5179-SK were transformed into oil palm protoplasts, respectively. **(C)** The relative expression level of *EgMADS16* in oil palm protoplasts that transformed either or both of *EgmiR5179* and *EgMADS16*. The values are the means ± SD (*n* = 3), “^*^” represents a significant difference (*p* < 0.05), and “^**^” represents a highly significant difference (*p* < 0.01) using Student’s *t*-test.

According to previous research, *OitaDEF2* is the target of *OitamiR5179* in *Orchid orchis italics* (Aceto et al., 2014). Likewise, *CenDEF3* is the target of *CenmiR5179* in *Cymbidium ensifolium* (Li et al., 2015). Therefore, small RNA target analysis (see footnote 3) suggested that *EgMADS16* is targeted by *EgmiR5179*. To verify this target relationship, we performed a dual-luciferase reporter assay in *Arabidopsis* and oil palm protoplasts. The results indicated that the relative luciferase activity of *EgMADS16*-LUC-transfected protoplasts was decreased with the expression of *EgmiR5179* (Figures 3A,B). At the same time, we transformed either or both EgmiR5179-SK and *EgMADS16*-LUC into oil palm protoplasts, and then, the relative expression level of *EgMADS16* was detected by qPCR. The results were consistent with the results of the dual-luciferase reporter assay in which the *EgMADS16* expression level was downregulated by *EgmiR5179* (Figure 3C).

### Effect of *EgMADS16* on Lipid Biosynthesis

**Figure 4:**
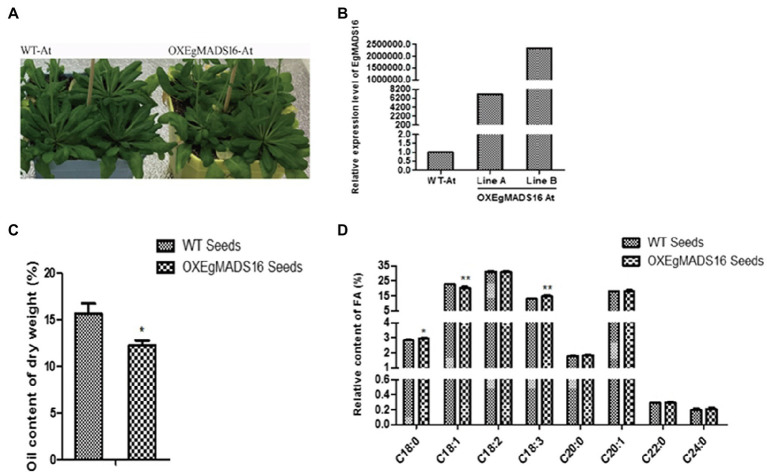
Effect of EgMADS16 on lipid biosynthesis in oil palm embryonic callus. **(A)** Wild-type oil palm (WT-OP) embryonic callus and *EgMADS16* overexpression oil palm embryonic callus (OXEgMADS16 OP) on screening medium containing glufosinate ammonium. Scale bar, 3 mm. **(B)** The relative expression level of *EgMADS16* in WT-OP and OXEgMADS16 OP. **(C)** The oil content of WT and OXEgMADS16 oil palm embryonic callus. **(D)** The relative FA content of WT and OXEgMADS16 oil palm embryonic callus. The values are the means ± SD (*n* = 3), “^*^” represents a significant difference (*p* < 0.05), and “^**^” represents a highly significant difference (*p* < 0.01) using Student’s *t*-test.

**Figure 5:**
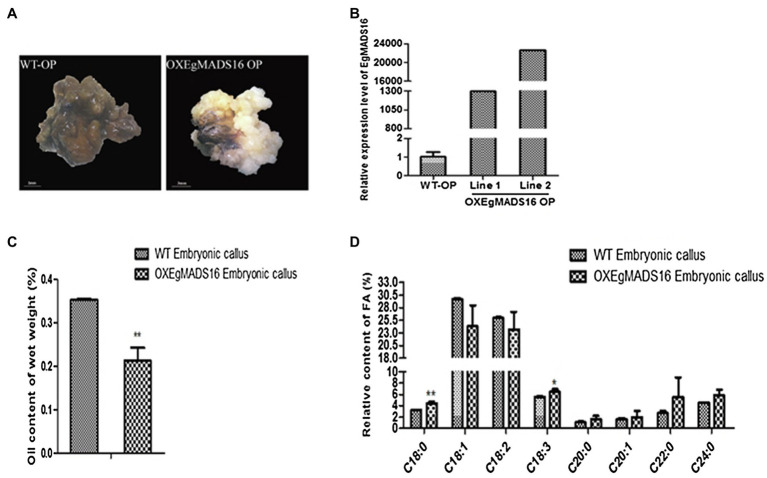
Effect of EgMADS16 on lipid biosynthesis in *Arabidopsis*. **(A)** Wild-type Arabidopsis (WT-At) and *EgMADS16* overexpression Arabidopsis (OXEgMADS16 At). **(B)** The relative expression level of *EgMADS16* in WT-At and OXEgMADS16 At. **(C)** The oil content of WT and OXEgMADS16 Arabidopsis seeds. **(D)** The relative FA content of WT and OXEgMADS16 Arabidopsis seeds. The values are the means ± SD (*n* = 3), “^*^” represents a significant difference (*p* < 0.05), and “^**^” represents a highly significant difference (*p* < 0.01) using Student’s *t*-test.

To characterize the role of *EgMADS16* in lipid biosynthesis, we obtained *EgMADS16*-overexpressing *Arabidopsis* lines (OX*EgMADS16* Line A and Line B; Figure 4A) and oil palm embryonic callus lines (OX*EgMADS16* Line 1 and Line 2; Figure 5A). Compared with the wild type, all OX*EgMADS16* lines had higher *EgMADS16* expression. Meanwhile, the relative fatty acid contents of OX*EgMADS16 Arabidopsis* seeds and oil palm embryonic calli were tested by GC. The results showed that the C18:0 and C18:3 contents of the OX*EgMADS16* lines significantly increased, while the C18:1 content decreased compared with that of the wild type (Figures 4D, 5D). In addition, there were significant decreases in the lipid contents of both OX*EgMADS16 Arabidopsis* seeds and oil palm embryonic calli (Figures 4C, 5C).

### Regulation of *EgMADS16* in Lipid Biosynthesis by *EgmiR5179*

**Figure 6:**
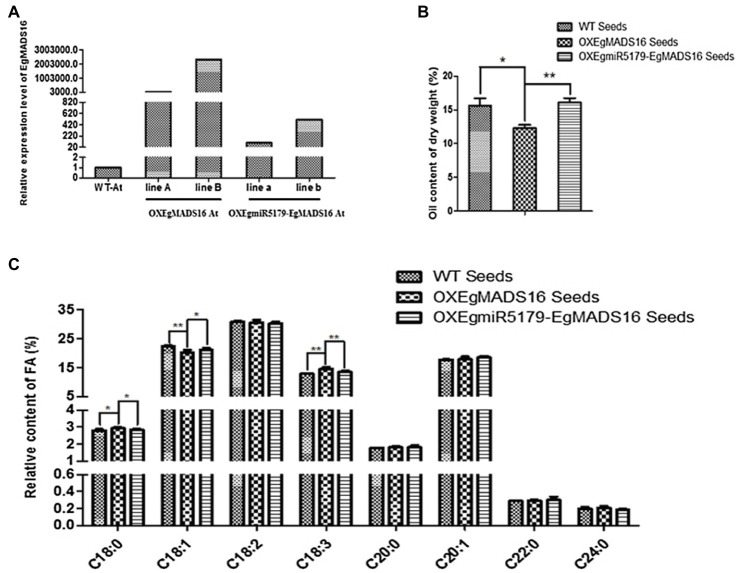
The effect of *EgMADS16* on lipid biosynthesis is inhibited by *EgmiR5179* in Arabidopsis. **(A)** The relative expression level of *EgMADS16* in wild-type and transgenic Arabidopsis. **(B)** The oil content of wild-type and transgenic Arabidopsis seeds. **(C)** The relative FA content of wild-type and transgenic Arabidopsis seeds. The values are the means ± SD (*n* = 3), “^*^” represents a significant difference (*p* < 0.05), and “^**^” represents a highly significant difference (*p* < 0.01) using Student’s *t*-test. OXEgmiR5179-EgMADS16 At: Arabidopsis coexpressed *EgmiR5179* and *EgMADS16*.

Based on the predicted targeting regulatory relationship, *EgmiR5179* should regulate the expression of *EgMADS16* and thus play a role in lipid biosynthesis. To verify this hypothesis, we generated transgenic *Arabidopsis* plants that coexpressed *EgmiR5179* and *EgMADS16* (OXEgmiR5179-*EgMADS16*; Figure 6A) and analyzed the FA and oil contents. As expected, *EgmiR5179* weakened the inhibitory effect of *EgMADS16* on oil contents (Figure 6B). Additionally, the relative contents of C18:0 and C18:3 in OXEgmiR5179-*EgMADS16* seeds decreased compared with those of the OX*EgMADS16* seeds, while those in OX*EgMADS16* seeds rose compared with WT seeds. The C18:1 content of OXEgmiR5179-*EgMADS16* seeds increased compared with that of OX*EgMADS16* seeds, while that of OX*EgMADS16* seeds decreased compared with that of WT seeds (Figure 6C).

### Identification of the Downstream Genes of *EgMADS16*

**Figure 7:**
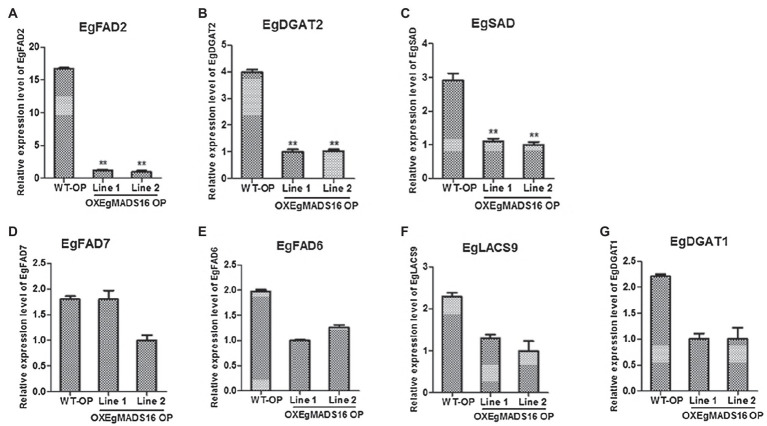
Identification of downstream genes of EgMADS16 protein. **(A–G)** The relative expression level of *EgFAD2* **(A)**, *EgDGAT2* **(B)**, *EgSAD* **(C)**, *EgFAD7* **(D)**, *EgFAD6* **(E)**, *EgLACS9* **(F)**, and *EgDGAT1* **(G)** in WT and OXEgMADS16 oil palm embryonic callus. The values are the means ± SD (*n* = 3). “^**^” represents a highly significant difference (*p* < 0.01) using Student’s *t*-test.

*EgMADS16* belongs to MADS-box family. This means that it is likely to function as a transcription factor to regulate lipid biosynthesis by regulating lipid biosynthesis-related genes. Therefore, we selected seven related genes according to the results of fatty acid compositions and contents of OXEgMADS lines, and we examined the expression level of these genes in OXEgMADS oil palm embryonic callus by qPCR. As a result, the expression levels of *EgFAD2*, *EgDGAT2*, and *EgSAD* significantly declined, while those of *EgFAD7*, *EgFAD6*, *EgLACS9*, and *EgDGAT1* did not significantly change (Figures 7A–G).

To further investigate whether *EgMADS16* protein regulates the expression of *EgFAD2*, *EgDGAT2*, and *EgSAD* by binding to their promoters, we conducted yeast one-hybrid assays. However, interactions between *EgMADS16* and the promoters of *EgFAD2, EgDGAT2*, or *EgSAD* were not detected (Supplementary Figure 1), suggesting that *EgMADS16* downregulated these genes by interacting with other DNA-binding transcription factors rather than directly binding to their promoters.

### *EgMADS16* Binds to EgGLO1 Proteins

**Figure 8:**
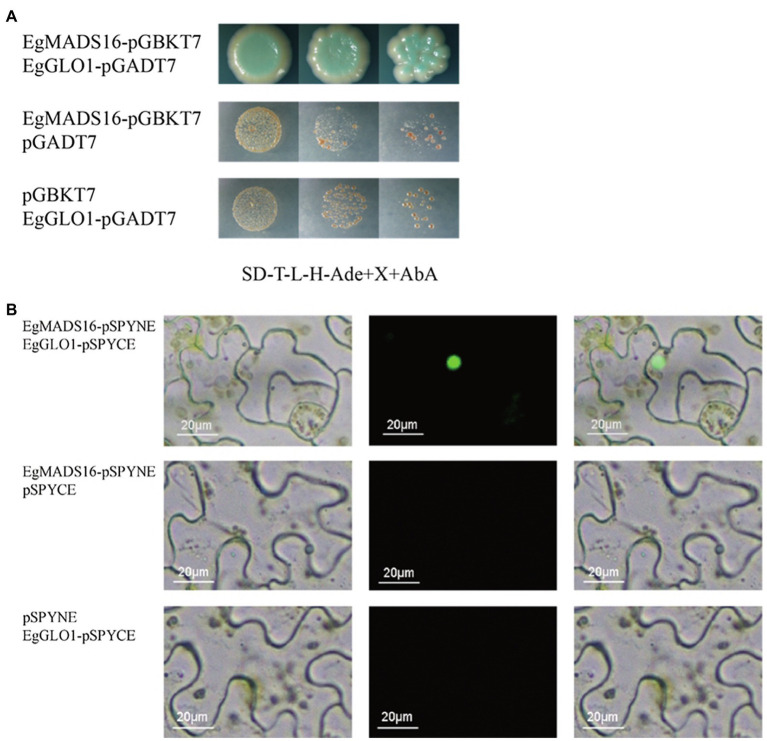
EgMADS16 protein binds to EgGLO1 protein. **(A)** Yeast two-hybrid assay of EgMADS16 protein and EgGLO1 protein. **(B)** BiFC of EgMADS16 protein and EgGLO1 protein. Scale bar, 20 μm.

Next, we used yeast two-hybrid assays to identify the transcription factors that interact with *EgMADS16* proteins. Considering the activation domains of transcription factors, we tested the autoactivation of *EgMADS16* on yeast reporters. The results showed that *EgMADS16* proteins have an activation domain, but Aba can inhibit its autoactivation (Supplementary Figure 2). Therefore, we performed yeast two-hybrid assays and found that there was an interaction between *EgMADS16* and EgGLO1 (XP_010911271.1) proteins (Figure 8A). To further verify this interaction, BiFC was used. YFP protein fluorescence was observed only when *EgMADS16*-pSPYNE and EgGLO1-pSPYCE were coinfiltrated into the leaves of *N. benthamiana* (Figure 8B), suggesting that *EgMADS16* binds to the EgGLO1 proteins.

## Discussion

In this paper, the role of the *EgMADS16* gene in oil biosynthesis was studied. Among the oil palm fruits at five different developmental stages, the *EgMADS16* gene had the highest transcription level in the fourth stage and decreased in the fifth stage. The phylogenetic tree analysis showed that PdMADS16 had the closest relationship with *EgMADS16*, followed by *AcMADS16* and *MaMADS16*, but their functions have not been reported. The subcellular localization results showed that *EgMADS16* protein was located in the nucleus, which is consistent with the localization of other transcription factors. Previous studies found that MADS-box genes were closely related to plant growth and development. In oil palm, it has been proven that the MADS-box gene *EgSQUA1* is related to the height of the plant and the length of pedicels and siliques; *EgGLO2* promotes a partial conversion of sepals to petals in whorl 1; and *EgAGL2-1* is involved in stamens and gynoecium development (Adam et al., 2007). However, the overexpression of *EgMADS16* (*EgDEF1*) did not change the phenotype of flowers (Adam et al., 2007) but participated in the biosynthesis of oil and fatty acids, which was demonstrated by the significant increases in C18:0 and C18:3 contents and the decreases in C18:1 content and total oil yield (Figures 4, 5).

As a transcription factor, *EgMADS16* likely participates in the regulation of oil biosynthesis in the same way. According to the fatty acid compositions and contents of OXEgMADS lines, *EgSAD*, *EgFAD2*, *EgFAD6*, *EgFAD7*, *EgLACS9*, *EgDGAT1*, and *EgDGAT2* were selected as candidate genes, and qRT-PCR was used to detect the expression levels of these seven oil biosynthesis-related genes in OX*EgMADS16* oil palm embryonic calli. As a result, the transcription levels of *EgSAD*, *EgFAD2*, and *EgDGAT2* were significantly suppressed (Figures 7A–C). This result indicates that *EgMADS16* regulates the contents of fatty acids and oil by inhibiting the expression of EgSAD, EgFAD2, and EgDGAT2. To determine whether the *EgMADS16* protein directly regulates the expression of these three genes by binding to their promoters, a yeast one-hybrid assay was conducted. However, the results showed that the *EgMADS16* protein cannot directly bind to the promoters of *EgSAD*, *EgFAD2*, and *EgDGAT2*. This means that *EgMADS16* protein likely inhibits the expression level of these three genes by interacting with an intermediate protein, which can directly bind to the promoters of *EgSAD*, *EgFAD2*, and EgDGAT2.

In addition, small RNA target analysis showed that *EgMADS16* is targeted by *EgmiR5179* (Supplementary Figure 3). Moreover, the dual-luciferase reporter assay and qRT-PCR in protoplasts further suggested this target relationship: *EgmiR5179* can inhibit the expression of *EgMADS16* (*EgDEF1*; Figure 3). The overexpression of *EgmiR5179* significantly increased the total oil content in seeds (Gao et al., 2019). In this study, the overexpression of *EgMADS16* significantly reduced the total oil content of *Arabidopsis* seeds and oil palm embryonic calli (Figures 4, 5), which is consistent with the role of *EgMADS16* as the target gene of *EgmiR5179*. In addition, compared with OXMADS16 transgenic *Arabidopsis* seeds, the total oil content of OXEgmiR5179-*EgMADS16* seeds increased significantly, C18:0 and C18:3 decreased, and C18:1 increased. More interestingly, this trend of change was opposite to the trend of oil and fatty acid contents in the seeds of OXMADS16 plants relative to the wild type (Figures 6B,C). This implies that EgmiR5179 can inhibit the regulation of *EgMADS16* in the biosynthesis of oil and FAs and promote the accumulation of oil.

In previous studies, it has been proven that homologous complexes formed between MADS-box proteins to facilitate the binding of MADS-box proteins to DNA. For example, OsMADS16 protein interacts with OsMADS4, OsMADS6, and OsMADS8 proteins to regulate stamen development (Lee et al., 2003); GmMADS28 protein interacts with SOC1, AP1, and AGL8/FUL proteins to modulate floral organ number, petal identity, and sterility (Huang et al., 2014). Similarly, the results of yeast two-hybrid assays and BiFC suggested that there was an interaction between the *EgMADS16* protein and EgGLO1 protein (Figure 8). *EgMADS16* proteins regulate oil biosynthesis by interacting with the EgGLO1 proteins, which directly bind to the promoters of oil biosynthesis-related genes. However, there is no interaction between the OsMADS16 protein and OsMADS2 (Lee et al., 2003), which are homologs of *EgMADS16* and EgGLO1. In addition, this may be due to species differences.

**Figure 9:**
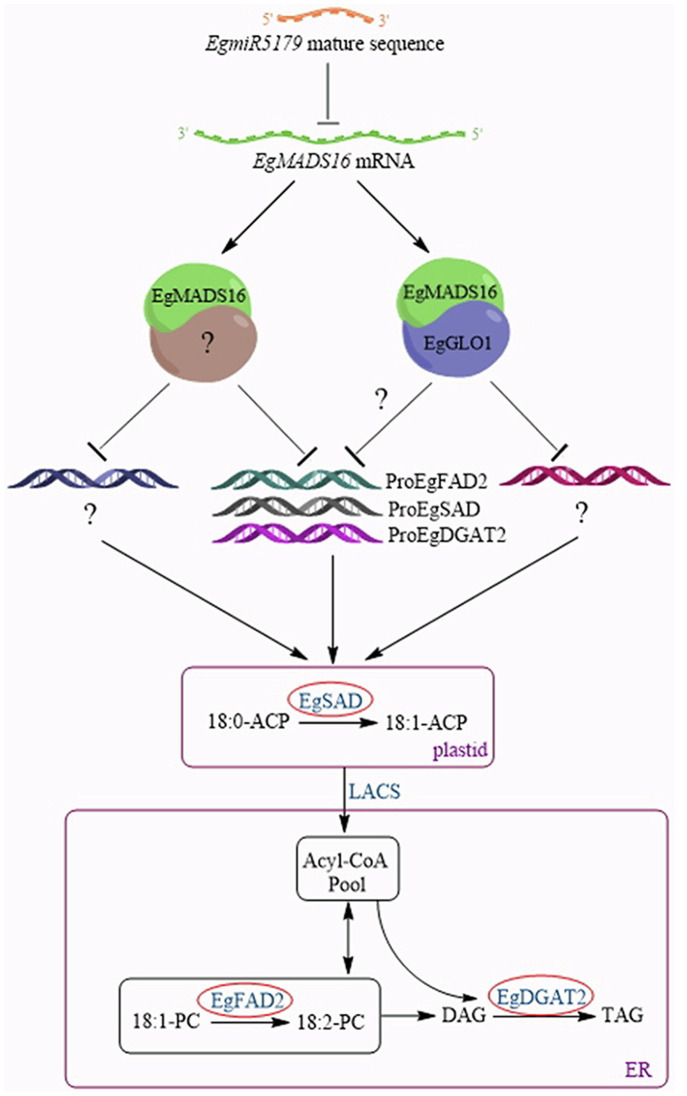
A model about the molecular mechanism of *EgMADS16* regulating oil biosynthesis. EgMADS16 protein forms a complex with EgGLO1 or other proteins and binds to the promoter of *EgFAD2* or *EgSAD* or *EgDGAT2* or other oil biosynthesis-related genes, inhibiting the transcription of these genes and affecting fatty acid components and oil accumulation. However, *EgmiR5179* targets to *EgMADS16* mRNA and inhibits its expression, so that the oil accumulation is promoted.

In summary, we are hypothesizing a model of the molecular mechanism by which *EgMADS16* regulates oil biosynthesis (Figure 9). After *EgMADS16* is translated, the *EgMADS16* protein forms a complex with EgGLO1 or other proteins and binds to the promoter of *EgFAD2, EgSAD, EgDGAT2*, or other oil biosynthesis-related genes, inhibiting the transcription of these genes and affecting fatty acid components and oil accumulation. However, when *EgmiR5179* is overexpressed, the *EgmiR5179* mature sequence binds to *EgMADS16* mRNA and inhibits its expression so that the transcription of downstream oil biosynthesis-related genes cannot be inhibited by *EgMADS16* protein.

Nevertheless, the intermediate proteins regulated by *EgMADS16* proteins for the transcription of *EgSAD*, *EgFAD2*, and *EgDGAT2* have yet to be studied. It is unclear which oil biosynthesis-related genes, the *EgMADS16*/EgGLO1 complex, regulate. Nevertheless, the molecular mechanism presented in this paper provides a preliminary understanding of the regulation of oil biosynthesis by transcription factors in oil palm, which lays the foundation for further research and provides a strategy for obtaining a higher yield of oil palm in the future.

## Data Availability Statement

The datasets presented in this study can be found in online repositories. The names of the repository/repositories and accession number(s) can be found in the article/Supplementary Material.

## Author Contributions

DL and YZ designed the research. YW, JZo, and JZh performed the research. YZ and DL wrote the paper. All authors read and approved the final manuscript.

## Conflict of Interest

The authors declare that the research was conducted in the absence of any commercial or financial relationships that could be construed as a potential conflict of interest.

## Publisher’s Note

All claims expressed in this article are solely those of the authors and do not necessarily represent those of their affiliated organizations, or those of the publisher, the editors and the reviewers. Any product that may be evaluated in this article, or claim that may be made by its manufacturer, is not guaranteed or endorsed by the publisher.

## Footnotes

^1^https://www.ncbi.nlm.nih.gov/Structure/cdd/wrpsb.cgi

^2^http://www.ebi.ac.uk/Tools/msa/clustalo/

^3^http://plantgrn.noble.org/psRNATarget/analysis?function=3

## Abbreviations

AP2: APETALA2
AS: acetosyringone
BiFC: bimolecular fluorescence complementation
BR: biological reagent
CDSs: coding region sequences
CoA: coenzyme A
DAP: days after pollination
DAPI: 4', 6-diamidino-2-phenylindole
DGAT: diacylglycerol acyltransferase
FA: fatty acid
FAD: fatty acid dehydrogenase
FAME: fatty acid methyl ester
GC: gas chromatography
GFP: green fluorescent protein
LACS: long-chain acyl-CoA synthetase
MS medium: Murashige and Skoog medium
OD: optical density
OX: over-expressed
PEG: polyethylene glycol
qPCR: quantitative real-time PCR
SAD: stearoyl-ACP desaturase
YFP: yellow fluorescent protein
