# Supplementary material for: EgmiR5179 Regulates Lipid Metabolism by Targeting EgMADS16 in the Mesocarp of Oil Palm (Elaeis guineensis)
Source: Front Plant Sci. 2021 Jul 26;12:722596. doi: 10.3389/fpls.2021.722596 (PMC8350733; doi:10.3389/fpls.2021.722596)
Supplement: Supplementary file 1 [file Data_Sheet_1.doc]

**"Table 1** qTR-PCR primers

| **Gene Name** | **Primer Name** | **Sequence（5′- 3′）** |
| --- | --- | --- |
| *EgMADS16* | EgMADS16-qPCR-F | CACGCAGACGGATACCTACAAG |
|  | EgMADS16-qPCR-R | GCTCGGCTGAACACGGAAA |
| *β-actin* | β-actin-qPCR-F | TGGAAGCTGCTGGAATCCAT |
|  | β-actin-qPCR-R | TCCTCCACTGAGCACAACGTT |
| *Actin7* | Actin7-qPCR-F | GCCCCTGAGGAGCACCCAGTT |
|  | Actin7-qPCR-R | CCGGTTGTACGACCACTGGCA |
| *EgSAD* | EgSAD-qPCR-F | GGCTTGGACTGCTGAAGAGAAC |
|  | EgSAD-qPCR-R | TGAAAGTCGCCCTCTCCTGAAA |
| *EgFAD2* | EgFAD2-qPCR-F | CCCCATTCACATTGAGCCA |
|  | EgFAD2-qPCR-R | GGGTTGTTGATGTACCTGGAAT |
| *EgFAD6* | EgFAD6-qPCR-F | TTGGTGGCACAGTCCATTGT |
|  | EgFAD6-qPCR-R | TGATGAGCTGCCTGCAAGTT |
| *EgFAD7* | EgFAD7-qPCR-F | CCGCCGCCTACCTCAATAACT |
|  | EgFAD7-qPCR-R | TGCCCAACCACGCTGTTCA |
| *EgDGAT1* | EgDGAT1-qPCR-F | AGTAGTGCTTATTGCGGTGAACAG |
|  | EgDGAT1-qPCR-R | TGAAGTGTGATAACAACTGGCTCAG |
| *EgDGAT2* | EgDGAT2-qPCR-F | CGATTGGCGTTGTTGCTCTC |
|  | EgDGAT2-qPCR-R | CCATGTCCACAAGTGCCTCA |
| *EgLACS9* | EgLACS9-qPCR-F | GCATCTGTTGGTCGTGTTGGT |
|  | EgLACS9-qPCR-R | CGAGGCATTGGTGAATCTGTGA |

**Table 2 Primers of v**ector construction

| **Gene Name** | **Primer Name** | **Sequence（5′- 3′）** |
| --- | --- | --- |
| *EgMADS16* | P3301-F | TTGGTACCATGGGGAGGGGGAAGATA |
|  | P3301-R | ATGGATCCTCAAGCAAGGCGCAGGT |
|  | LUC-F | CATCTAGAATGGGGAGGGGGAAGATA |
|  | LUC-R | ATGGATCCTCAAGCAAGGCGCAGGT |
|  | p1300S GFP-F | TACAAATCTATCTCTGGATCCATGGGGAGGGGGAAGATA |
|  | p1300S GFP-R | GCTCACCATGGTGGCGGATCCAGCAAGGCGCAGGT |
|  | pGADT7-F | GCCATGGAGGCCAGTGAATTCATGGGGAGGGGGAAGATA |
|  | pGADT7-R | GCAGCTCGAGCTCGATGGATCCTCAAGCAAGGCGCAGGT |
|  | pGBKT7-F | AGGCCGAATTCCCGGGGATCCATGGGGAGGGGGAAGATA |
|  | pGBKT7-R | CCGCTGCAGGTCGACGGATCCTCAAGCAAGGCGCAGGT |
|  | pSPYNE-F | GAGAACACGGGGGACTCTAGAATGGGGAGGGGGAAGATA |
|  | pSPYNE-R | GACAGTACTATCGATGGATCCAGCAAGGCGCAGGT |
| *EgGLO1* | pSPYCE-F | GAGAACACGGGGGACTCTAGAATGGGGCGAGGGAAGATT |
|  | pSPYCE-R | GACAGTACTATCGATGGATCCATTGTTCTCTTGCA |
