# Supplementary figures and images for: EgmiR5179 Regulates Lipid Metabolism by Targeting EgMADS16 in the Mesocarp of Oil Palm (Elaeis guineensis)
Source: Front Plant Sci. 2021 Jul 26;12:722596. doi: 10.3389/fpls.2021.722596 (PMC8350733; doi:10.3389/fpls.2021.722596)

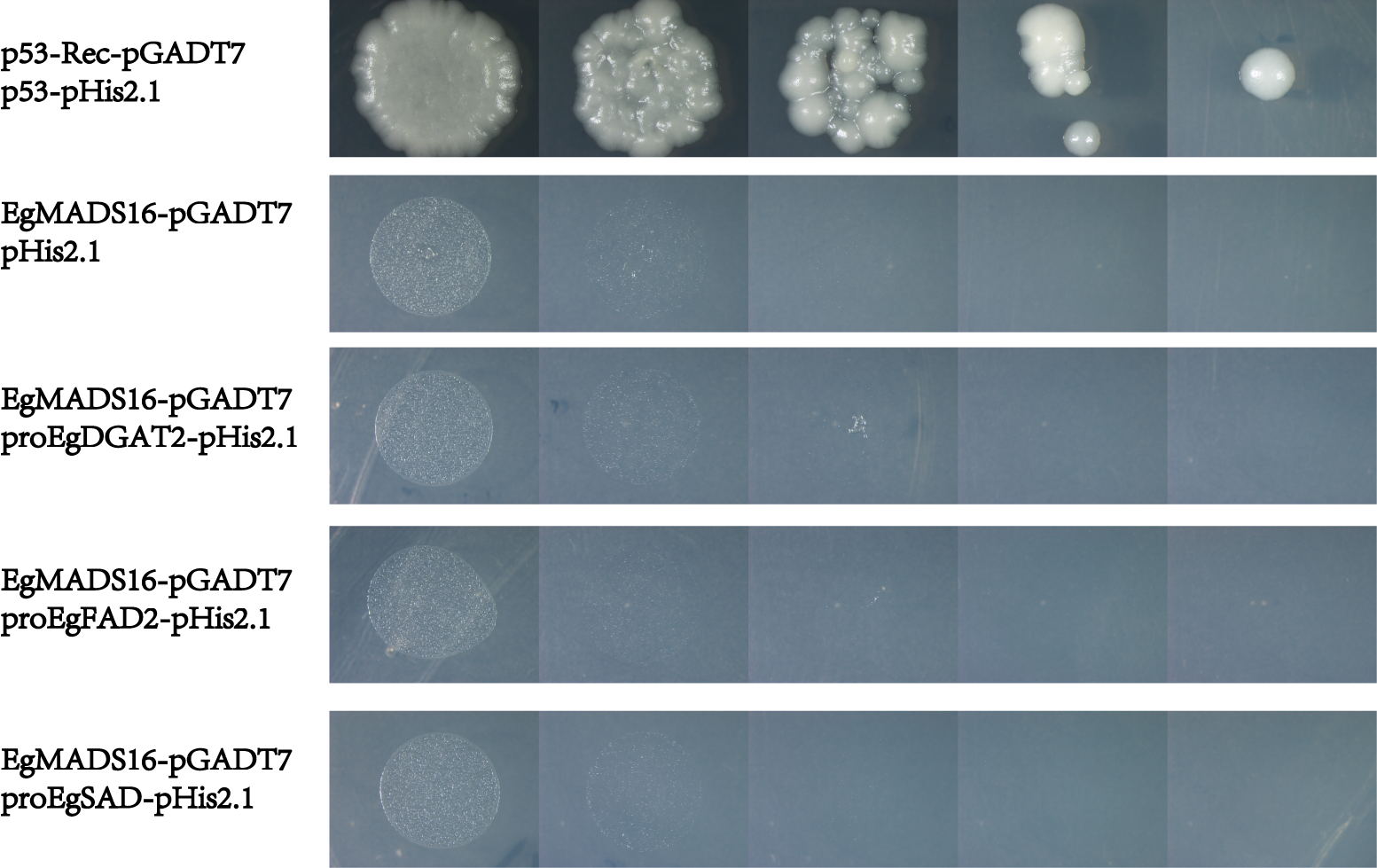

Supplement: Supplementary file 2 [file Image_1.JPEG]

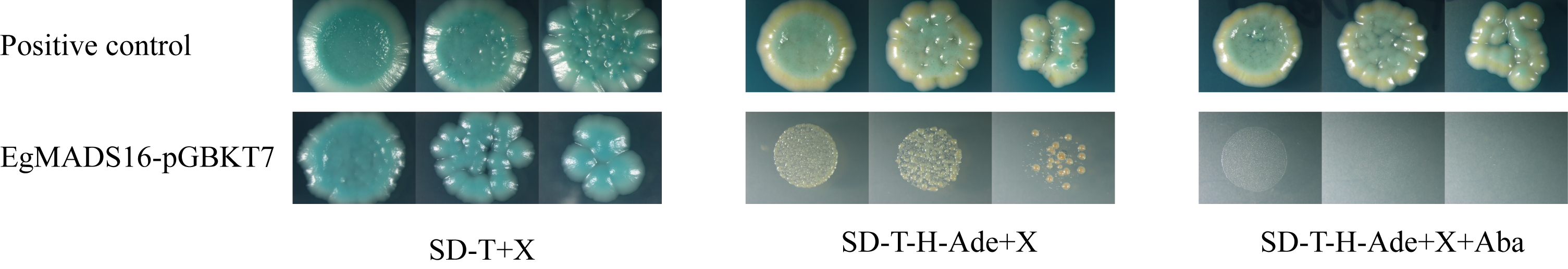

Supplement: Supplementary file 3 [file Image_2.JPEG]

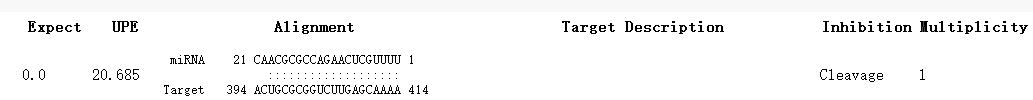

Supplement: Supplementary file 4 [file Image_3.JPEG]
